# Telomere length, cardiovascular risk and arteriosclerosis in human kidneys: an observational cohort study

**DOI:** 10.18632/aging.100814

**Published:** 2015-09-30

**Authors:** Katrien De Vusser, Nicky Pieters, Bram Janssen, Evelyne Lerut, Dirk Kuypers, Ina Jochmans, Diethard Monbaliu, Jacques Pirenne, Tim Nawrot, Maarten Naesens

**Affiliations:** ^1^ Department of Microbiology and Immunology, KU Leuven – University of Leuven, Leuven, Belgium; ^2^ Department of Nephrology and Renal Transplantation, University Hospitals Leuven, Leuven, Belgium; ^3^ Centre for Environmental Sciences, Hasselt University, Hasselt, Belgium; ^4^ Department of Imaging and Pathology, KU Leuven – University of Leuven, Leuven, Belgium; ^5^ Department of Pathology, University Hospitals Leuven, Leuven, Belgium; ^6^ Department of Abdominal Transplantation Surgery, University Hospitals Leuven, Leuven, Belgium; ^7^ Department of Public Health and Primary Care, KU Leuven – University of Leuven, Leuven, Belgium

**Keywords:** telomere length, replicative senescence, arteriosclerosis, kidney, histology

## Abstract

**Background:**

Replicative senescence, associated with telomere shortening, plays an important role in aging and cardiovascular disease. The relation between telomere length, cardiovascular risk, and renal disease is unknown.

**Methods:**

Our study consisted of a cohort of 257 kidney donors for transplantation, divided into a test and a validation cohort. We used quantitative RT‐PCR to measure relative telomere length (log T/S ratio) in peripheral blood leucocytes, and in kidney biopsies performed prior to implantation. The association between leucocyte and intrarenal telomere length, cardiovascular risk factors, and renal histology, was studied using multiple regression models, adjusted for calendar age, gender and other donor demographics.

**Results:**

Subjects with intrarenal arteriosclerosis had significantly shorter leucocyte telomere length compared with patients without arteriosclerosis (log T/S ratio ‐0.3 ± 0.4 vs. 0.1 ± 0.2 with vs. without arteriosclerosis; *p* = 0.0008). Intrarenal arteriosclerosis was associated with shorter telomere length, independent of gender, calendar age, history of hypertension and history of cardiovascular events. For each increase of one standard deviation of the log T/S ratio, the odds for intrarenal arteriosclerosis decreased with 64% (Odds ratio 0.36; 95% CI 0.17‐0.77; *p* = 0.02). In accordance with leucocyte telomere length, shorter intrarenal telomere length associated significantly with the presence of renal arteriosclerosis (log T/S ratio ‐0.04 ± 0.06 vs. 0.08 ± 0.01 with vs. without arteriosclerosis, *p* = 0.007), and not with other histological lesions.

**Interpretation:**

We demonstrate that arteriosclerosis in smaller intrarenal arteries is associated with shorter telomere length. Our study suggests a central role of replicative senescence in the progression of renovascular disease, independent of calendar age.

## INTRODUCTION

Cellular or replicative senescence, associated with telomere shortening, is considered as one of the most important drivers of aging [1]. This phenomenon leads to permanent and irreversible growth arrest [2]. Telomeres comprise tandem TTAGGG repeats of 5000 to 15000 base pairs that reside at the ends of chromosome ends. Their main function is to cap these chromosome ends and prevent chromosomal instability [3]. Although telomere length is regulated by telomerase, telomeres tend to progressively shorten with every cell division [4, 5]. Although telomere length is partly heritable [6], there are major differences in telomere length even among monozygotic twins, which illustrates that environmental factors play a major role in the rate of telomere attrition. Due to their high content of guanines, telomeres are highly sensitive to oxidative damage [7]. Accelerated shortening of telomeres or senescence of cells is an important pathway by which oxidative stress accelerates biological aging and age-related diseases like atherosclerosis and cardiovascular disease [8, 9]. Obesity and smoking are associated with leucocyte telomere attrition, as well as hypertension, insulin resistance and diabetes, male gender and lower socioeconomic status [6, 10–16].

In contrast to studies that evaluated the determinants of telomere shortening and association with disease risk, the relation between telomere shortening and organ histology was only studied scarcely. In kidneys, the histology and function deteriorates with increasing age. Animal studies and *in vitro* data suggested that replicative senescence plays a crucial role in this deterioration of renal histology and function [17]. These findings have however not been validated in humans, except for a small study where intrarenal telomere length did not associate with renal histology [18]. Moreover, the association of telomere length with non-diseased organ histology has not been evaluated in other organ systems, despite the central role of telomere shortening and replicative senescence in aging. Finally, the correlation between telomere length measured in organ tissue, and peripheral blood leucocyte telomere length remains currently unclear. Therefore, we evaluated the association between renal histology, leucocyte and intrarenal telomere length, cardiovascular risk factors and calendar age, in a cohort of kidney donors for transplantation.

## RESULTS

### Population characteristics

Cohort 1 consisted of 217 unique kidney donors with sufficient amount of good-quality leucocytic DNA available for evaluation of telomere length. In this cohort, 144 pre-implantation kidney biopsies were available for histological evaluation. Fourteen biopsies were of insufficient quality according to the Banff 1997 criteria, leaving 130 baseline biopsies for histological analysis. On average, 24.0 ± 16.7 glomeruli were obtained per biopsy (range, 10–89).

Cohort 2, for evaluation of intrarenal telomere length, consisted of 40 kidney donors. Of these 40 subjects, good-quality DNA from leucocytes was available for 32 subjects and good-quality DNA derived from biopsies was available for all 40 kidneys. All 40 biopsies included in this cohort were of sufficient quality according to the Banff 1997 criteria for histological evaluation.

Table 1 summarizes the characteristics of these two cohorts and the histology of the biopsies that were included. Kidney function, expressed as eGFR, was 89.7 ± 40.9 (range 42.3–189.3) mL/min/1.73m^2^ in Cohort 1 and 114 ± 42.0 (range 60.8–197.2) mL/min/1.73m2 in Cohort 2, respectively.

**Table 1:** Demographics and histology the subjects and biopsies included in this study

	*Cohort 1*	*Cohort 2*
N	*217*	*40*
Leucocyte Telomere Length (log T/S ratio)	0.05 ± 0.20	0.07 ± 0.09^&^
Intrarenal Telomere Length (log T/S ratio)	-	−0.08 ± 0.12
Calendar Age (years)	44.4 ± 15.0	48.1 ± 15.0
Male Gender % (N)	55.3% (120)	57.5%
Deceased Donor % (N)	97.7% (212)	90.0% (36)
Brain Death / Cardiac Death	86.8%(184)/13.2%(28)	72.2%(26)/33.3%(10)
Ischemic Stroke as Reason for Death % (N)	3.2% (7)	10% (4)
Hemorrhagic Stroke as Reason for Death % (N)	43.8% (95)	22.5% (9)
History of Hypertension % (N)	24.7% (44/178)	37.5%
History of Diabetes Mellitus % (N)	1.8% (4)	8.3% (3)
History of Smoking % (N)	30.5% (54/177)	37.5%
Body Mass Index (kg/m2)	24.9 ± 5.5	25.6 ± 5.5
History of Cardiovascular Events % (N)	13.9% (14)	12.5% (5)
Serum Creatinine (mg/dl)	0.89 ± 0.3	0.88 ± 0.23
eGFR (mL/min/1.73m^2^)#	89.7 ± 40.9	114.0 ± 42.0
Renal Function (1/creatinine)	1.4 ± 0.8	1.2 ± 0.4
Presence of Arteriolar Hyalinosis % (N)*	30.8% (40/130)	10.0% (4)
Presence of Interstitial Fibrosis % (N)*	25.4% (33/130)	27.5%
Presence of Tubular Atrophy % (N)*	50.0% (65/130)	62.5% (25)
Presence of Arteriosclerosis % (N)*	7.7% (10/130)	10.0% (4)
Presence of > 10% Glomerulosclerosis% (N)*	15.4% (20/130)	20.0% (8)

Data are expressed as mean ± standard deviation unless otherwise specified;

& N = 30

# eGFR was calculated using the MDRD formula [19, 35].

### Calendar age, clinical demographics and leucocyte telomere length

Mean log T/S ratio of telomere length was 0.05 ± 0.25 (range −0.95 to 0.71). Leucocyte telomere length inversely correlated with older calendar age (r=−0.2, *p* = 0.005) (Table 2).

**Table 2:** Clinical determinants of leucocyte telomere length (log T/S) (N = 217)

	Univariate linear regression			Multivariate linear regression		
Parameter	Parameter estimate	Standard Error	P value	Parameter estimate	Standard Error	P value
Donor age (years)	−0.01	0.001	0.005	−0.003	0.002	0.02
Heart Beating Donor/Non Heart Beating Donor*	−0.01	0.05	0.85			
Living Donor/Deceased Donor*	−0.12	0.12	0.29			
Ischemic Stroke as Reason for Death*	0.15	0.1	0.12			
Hemorrhagic Stroke as Reason for Death*	0.02	0.03	0.57			
History of Hypertension	−0.12	0.04	0.009	−0.13	0.06	0.02
History of Diabetes Mellitus*	−0.01	0.003	0.44			
History of Smoking*	0.01	0.04	0.91			
Body Mass Index (kg/m2) *	−0.002	0.003	0.44			
History of Cardiovascular Events	−0.13	0.07	0.06	−0.21	0.09	0.01
eGFR (mL/min/1.73m2)*	0.00	0.00	0.58			
Gender (Female)	0.08	0.04	0.02	0.1	0.04	0.02

Univariate analyses were assessed by linear regression. Multiple linear regression, with backward parameter selection, was used to model the determinants of telomere length. Multivariate analyses presented in this table were performed on 177 subjects, as history of hypertension and smoking status were not available in 40 cases. When we included all 217 subjects of Cohort 1 in the multivariate models, by excluding the covariate history of hypertension from the analysis, the independent associations between telomere length and donor age (*p* = 0.003), history of cardiovascular events (*p* = 0.02), and gender (*p* = 0.002) remained statistically significant.

* These parameters were included in the multivariate models, but were not retained in the final model after backward parameter selection.

** Effect sizes (SE) express the change in log T/S ratio associated with given changes in parameters.

History of hypertension *vs.* no hypertension and history of cardiovascular events vs. no history also associated with shorter telomere length. Women had longer telomere length. Other clinical demographics, including history of diabetes mellitus, history of smoking, living versus deceased donation, brain death vs. cardiac death, ischemic and hemorrhagic stroke as reason for donor death, body mass index, and eGFR were not associated with leucocyte telomere length. In multivariate linear regression analysis, older calendar age, history of cardiovascular events, male gender and history of hypertension were independent explanatory factors for shorter telomere length (Table 2).

### Leucocyte telomere length, clinical demographics and renal histology

**Figure 1:**
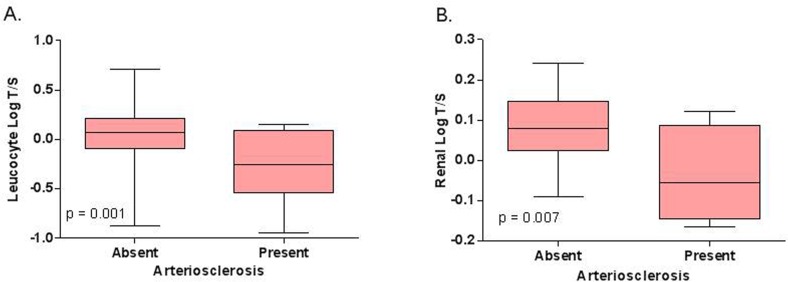
Relation between **(A)** leucocyte telomere length and renal arteriosclerosis in Cohort 1 (*N* = 130) and between **(B)** intrarenal telomere length and renal arteriosclerosis in Cohort 2 (*N* = 40). The p‐values represent nonparametric ANOVA. The horizontal lines within the boxes indicate means, the upper and lower ends of the boxes indicate standard deviations, and the whiskers indicate 95th percentiles.

In Cohort 1, arteriosclerosis in renal biopsies significantly associated with shorter leucocyte telomere length (log T/S ratio −0.3 ± 0.4 vs. 0.1 ± 0.2 in subjects with vs. without renal arteriosclerosis; *p* = 0.0008 (Figure 1B and Table 3). For one standard deviation increase of the log T/S ratio, independent of calendar age, gender and history of cardiovascular event, the odds of renal arteriosclerosis decreased by 64% (Odds ratio 0.36; 95% CI 0.17–0.77; *p* = 0.02). Moreover, arteriosclerosis in renal biopsies significantly associated with Δ biological age - calendar age (*r* = 0.3; *p* = 0.005).

**Table 3:** Association between the histological lesions in kidney biopsies, leucocyte telomere length and calendar age (*N* = 130)

		Telomere length*	Calendar age^°^
**Arteriolar hyalinosis**	Univariate	O*R* = 0.90 (0.61–1.34); p= 0.61	O*R* = 1.48 (1.14–1.97); p= 0.02
	Multivariate	ns	O*R* = 1.49 (1.07–2.08); p= 0.02
**Interstitial Fibrosis**	Univariate	O*R* = 0.90 (0.60–1.34); *p* = 0.58	O*R* = 1.79 (1.22–2.37); p= 0.0002
	Multivariate	ns	O*R* = 1.79 (1.23–2.62); p= 0.002
**Tubular Atrophy**	Univariate	O*R* = 0.89 (0.62–1.28); p= 0.54	O*R* = 1.97 (1.48–2.84); *p* < 0.0001
	Multivariate	ns	O*R* = 2.08 (1.48–2.94); *p* < 0.0001
**Arteriosclerosis**	Univariate	O*R* = 0.36 (0.32–0.77); p= 0.008	O*R* = 3.70 (1.22–12.8); p= 0.03
	Multivariate	O*R* = 0.36 (0.17–0.77); *p* = 0.02	ns
**Glomerulosclerosis**	Univariate	O*R* = 0.93 (0.56–1.51); p= 0.77	O*R* = 1.63 (1.10–2.37); p= 0.02
	Multivariate	ns	O*R* = 1.63 (1.10–2.59); *p* = 0.0006

Univariate and multiple logistic regression analyses were used to model the determinants of the different histological lesions. In multivariate analysis, we included calendar age and telomere length and adjusted for history of cardiovascular events and gender. When we also included the covariate history of hypertension in the analysis multivariate analysis (*N* = 109), the independent association between telomere length and renal arteriosclerosis (Odds ratio 0.34; 95% CI 0.17‐0.93; *p* = 0.03) remained statistically significant.

* Odds ratios for telomere length are calculated per increase of 1 standard deviation of log T/S ratio.

° Odds ratios for calendar age are given per 10 years increase. ns = not significant

Interstitial fibrosis, tubular atrophy, glomerulosclerosis and arteriolar hyalinosis did not associate with leucocyte telomere length. These histological lesions associated significantly with calendar age: for each 10 years increase in calendar age, the odds for interstitial fibrosis, tubular atrophy and glomerulosclerosis increased by 79%, 208% and 63%, respectively (Table 3).

**Figure 2:**
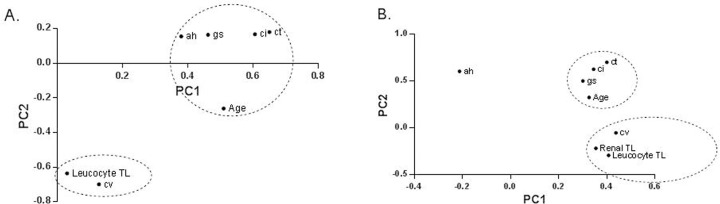
Principal component analysis using the individual histological lesions in kidney biopsies and telomere length in **(A)** Cohort 1 in **(B)** Cohort 2. This two‐dimensional scatter represents the histologic lesions according to their score in the loading matrix of a principal components analysis that included the different histological lesions, together with calendar age and telomere length. These analyses illustrated a dichotomy between the histological lesions associated with calendar age and the histological lesions associated with biological age. Calendar age (Age) are glomerulosclerosis (gs), tubular atrophy (ct), interstitial fibrosis (ci) and arteriolar hyalinosis (ah). Arteriosclerosis lesions (cv) clustered with biological age [leucocyte and renal telomere length (TL; log T/S ratio)]. PC1 = Principal Component 1; PC2 = Principal Component 2.

To further describe the complex relations between calendar age, leucocyte telomere length and renal histology, principal component analysis was performed. The summery plot is presented in Figure 2A. This analysis illustrated a dichotomy in the correlation between the histological findings and telomere length versus calendar age. Glomerulosclerosis, tubular atrophy, interstitial fibrosis and arteriolar hyalinosis clustered with calendar age, while arteriosclerosis clustered with leucocyte telomere length.

### Intrarenal telomere length, clinical demographics and renal histology

In Cohort 2, mean T/S ratio of intrarenal telomere length was 0.07 ± 0.09 (range −0.16–0.24). Intrarenal telomere length correlated significantly with leucocyte telomere length (*r* = 0.4, *p* = 0.001). Shorter intrarenal telomere length associated with history of hypertension (log T/S ratio 0.04 ± 0.02 vs. 0.10 ± 0.02 with vs. without hypertension; *p* = 0.05), but not with other demographics. Multivariate linear regression analysis was not performed in this smaller cohort to avoid overfitting of the data.

In this cohort, shorter intrarenal telomere length associated significantly with the presence of arteriosclerosis (log T/S ratio −0.04 ± 0.06 vs. 0.08 ± 0.01 with vs. without arteriosclerosis, *p* = 0.007) (Figure 1B). Intrarenal telomere length did not associate with any other histological parameter.

Principal component analysis was applied to further describe the complex relation between calendar age, renal histology, leucocyte telomere length and renal telomere length. The summery plots are presented in Figure 2B. This independent analysis confirmed the dichotomy in the correlation between the histological findings and telomere length versus calendar age, and the significant association between intrarenal telomere length and presence of renal arteriosclerosis.

## DISCUSSION

Our analysis of a cohort of prospectively collected native kidney biopsies illustrates that shorter telomere length (biological aging) associates with the presence of renal arteriosclerosis, but not with changes in the glomerular or tubulo-interstitial renal compartments. This association was independent of calendar age, gender, history of hypertension, and history of cardiovascular events, demographics all associated with telomere length. Our findings using leucocyte telomere length were confirmed in an independent validation cohort where telomere length was measured in intrarenal tissue.

The current study illustrated that intrarenal arteriosclerosis is associated with telomere length, independent of cardiovascular risk factors, history of cardiovascular disease and calendar age. Moreover, accelerated aging, calculated as the difference between biological age (telomere length) and calendar age associated highly significantly with intrarenal arteriosclerosis. This suggests a central role of telomere shortening or biological aging in the development renal arteriosclerosis and intimal hyperplasia. This finding could explain the previously observed association between shorter telomere length and clinical cardiovascular disease, also present in our study [8, 12, 25].

Intimal hyperplasia is the universal response of a vessel to injury, like repeated hemodynamic stress on the endothelium. The increased rate of cell turnover in regions with hemodynamic stress has been associated with accelerated telomere attrition and with endothelial cell senescence, which further contributes to endothelial dysfunction and intimal hyperplasia [26–29]. Moreover the senescence associated secretory phenotype of senescence cells causes a full range of autocrine and paracrine activities, aimed at tissue repair but also fueling degenerative and proliferative changes contributing to arteriosclerosis [8]. Injury-induced intimal hyperplasia underlies the pathogenesis of major cardiovascular diseases [30]. Next to hemodynamic stress that could lead to telomere attrition and intimal hyperplasia, also oxidative stress could be implicated in the pathogenesis of senescence-associated intimal hyperplasia. Oxidative damage is repaired less well in telomeric DNA than elsewhere in the chromosome, which leads to accelerated telomere loss [31]. On the basis of animal studies, a role for reactive oxygen species in intimal hyperplasia has been proposed [30]. Taken together, these data provide an explanation for the association we observed between shorter telomere length and renal arteriosclerosis (intimal hyperplasia).

In addition, the current study confirmed our previous data showing that older calendar age associates with chronic injury in the tubulointerstitial (tubular atrophy and interstitial fibrosis) and glomerular (glomerulosclerosis) renal compartments, while cardiovascular risk factors, cardiovascular disease and telomere length did not associate with these lesions [32]. It could therefore be hypothesized that these age-associated lesions represent accumulation of events over time that contribute to renal injury and thus represent a very different etiology. In contrast, senescence could be regarded as a more specific process that represents oxidative stress, endothelial dysfunction and arteriosclerosis and therefore contributes to the pathogenesis of cardiovascular disease. The differential association of calendar age versus telomere length with respectively glomerulosclerosis and tubulo-interstitial injury, versus renal arteriosclerosis, illustrates a robust dichotomy within the phenotype of “nephrosclerosis”, the primary structural findings of the aging kidney on light microscopy.

Moreover, we confirmed an association between hypertension, history of cardiovascular disease and shorter telomere length [11, 12, 33]. These associations do not prove causality. On one hand, telomere attrition and cardiovascular disease could be caused by common risk factors (smoking, hypertension, high total cholesterol level, obesity, physical inactivity), which contribute to inflammation and oxidative stress, and from this to accelerated telomere attrition [12]. On the other hand, hypertension could increase hemodynamic stress and herewith associated telomere attrition [28]. Finally, a prior study in a telomerase knockout mice model illustrated that short telomere length could even be the cause of hypertension, rather than its consequence [26].

Our study has some limitations. Although donor clinical demographics were collected prospectively, some kidney donor parameters that could be of importance could have been recorded incompletely. This could explain the lack of association between telomere shortening and smoking in our study, and between telomere shortening and diabetes mellitus, as was described previously [10, 15]. The fact that the number of patients with histological lesions was relatively small in this cohort, indicates that we might not have had precision in our point estimates. Finally, it has to be acknowledged that the reproducibility of semiquantitative histologic scoring of kidney biopsies is moderate at best, and that sampling error is inherent to any biopsy study. For this reason, all biopsies were obtained in a standardized fashion (wedge biopsies), and one single pathologist rescored all biopsies in batch, blinded for any clinical information. Wedge biopsies mostly include outer cortex, the zone where glomerulosclerosis and fibrosis are most severe. Arteriosclerosis, in contrast, most prominently affects larger arteries and therefore could be under-represented in a wedge biopsy [34]. Despite these shortcomings, the independent validation of the association between telomere length and renal arteriosclerosis supports the robustness and reproducibility of our data.

In conclusion, shorter leucocyte and intrarenal telomere length associated with renal arteriosclerosis, but not with other histological changes observed in older kidneys, like glomerulosclerosis or tubulo-interstitial injury. Our study therefore suggests a central role of replicative senescence in the progression of renovascular disease in humans. This association represents a missing link between cell and animal model studies associating telomere attrition with cardiovascular risk factors, and clinical studies associating telomere attrition with cardiovascular end points. Further study is necessary to elucidate whether telomere attrition plays a causal role in renal arteriosclerosis or is merely a sensor of other factors that lead to arteriosclerosis, and whether telomere attrition associates with histology of arteriosclerosis also in other organs.

## METHODS

### Inclusion and exclusion criteria

All prospective deceased donors of a single kidney transplant in adult recipients, which were performed between March 2004 and December 2010 at the University Hospitals Leuven (Leuven, Belgium), were eligible for Cohort 1 of this study. Since March 2004, kidney grafts undergo routine biopsies prior to implantation (*N* = 352). In Cohort 2, donor kidneys transplanted after November 2012 were biopsied prior to implantation, and were used for evaluation of intrarenal telomere length (*N* = 40). This study was approved by the Ethics Committee/Institutional Review Board of the University Hospitals Leuven, Leuven, Belgium (OG032; ML7499 and ML9785; clinicaltrials.gov NCT01331668).

### Clinical data collection

Clinical data were obtained from the Eurotransplant database (“Eurotransplant Donor Report”), which is maintained prospectively and is the central source of donor data for organ transplantation in the Eurotransplant region (www.donordata.eu). The following data were collected: calendar age, gender, cause of death, weight and length, living vs. deceased donor, brain death vs. cardiac death donor, body mass index, history of hypertension, diabetes mellitus, smoking, history of cardiovascular events prior to donation (including reason for death in deceased donors) and terminal serum creatinine levels before organ recovery. Renal function was estimated by the 4-variable Modification of Diet in Renal Disease (MDRD) equation (estimated glomerular filtration rate; eGFR) [19].

### Kidney biopsies and histologic evaluation

One pathologist (EL) reviewed all pre-implantation kidney biopsies, without knowledge of any demographic information of the kidney donor. The biopsy specimens were wedge biopsies with slides containing 4 to 10 paraffin sections (2 μm) that were stained with hematoxylin eosin, periodic acid–Schiff, and a silver methenamine staining method (Jones). The severity of histologic lesions, interstitial fibrosis (Banf “ci” score), tubular atrophy (Banf “ct” score), arteriolar hyalinosis (Banf “ah” score) and arteriosclerosis (Banf “cv” score = vascular fibrous intimal thickening = intimal hyperplasia), were scored semiquantitatively according to the Banff criteria [20]. In addition, the total number of glomeruli in each biopsy, and the number of globally sclerosed glomeruli, were calculated separately. Only biopsies with > 10 glomeruli (A quality) were included for evaluation of glomerulosclerosis.

### Telomere length in peripheral blood leucocytes

Peripheral blood samples for DNA extraction from leucocytes were routinely obtained from the donors at time of organ recovery. DNA was extracted using the simple salting out procedure for extracting DNA from human nucleated cells as described by S.A. Miller.21 Both DNA yield (ng/μL) and purity ratios A260/280 and A260/230 were determined and needed to be within strict quality limits (yield 50 ng/μL; purity ratio range 1.5–2 and 1.5–2 for A260/280 and A260/230, respectively) for inclusion of the samples in the study. Extracted DNA samples were stored at −80°C until further use. Leucocyte telomere length was measured as the telomere repeat copy number relative to two single gene nuclear control genes (*36B4* and *ACTB*), by a modified version of a previously described PCR based assay [20, 22]. The forward and reverse primers were 5′-ACACTAAGGTTTGGGTTTGGGTTTGGGTTTG GGTTAGTGT-3′ and 5′-TGTTAGGTATCCCTATC CCTATCCCTATCCCTATCCCTAACA-3′, respectively. The forward and reverse primers for the reference genes were respectively 5′-ACTCTTCCAGCCTTCCT TCC-3′ and 5′-GGCAGGACTTAGCTTCCACA-3′ for *ACTB,* and 5′-GGAATGTGGGCTTTGTGTTC-3′ and 5′-CCCAATTGTCCCCTTACCTT-3′ for *36B4*. The telomere reaction contained Fast SYBR^®^ Green I dye 2x (Applied Biosystems, Lennik, BE) mastermix, forward (100 nM) and reverse (900 nM) primer and 12.5 ng DNA. The telomere reactions were performed in triplicate. The thermal cycling profile for the telomere reaction consisted of following steps: 20 sec at 95°C, 2 cycles of 15 sec at 94°C and 15 sec at 49°C and 40 cycles of 15 sec at 94°C, 10 sec at 62°C and 15 sec at 74°C. For the reference genes a 10 μl PCR reaction contained Fast SYBR^®^ Green I dye 2x (Applied Biosystems, Lennik, BE) mastermix, forward (10 μM) and reverse (10 μM) primer and 12.5 μg DNA. Reactions were run in duplicate. Amplification specificity and absence of primer dimers was confirmed by melting curve analysis at the end of each run.

Each PCR-plate contained two inter-run calibrators and two no-template controls. For the telomere assay, reference sample were included, one with relative short telomeres and one with relative long telomere. After thermal cycling, raw data were collected and processed. Cq-values of telomere were normalized relative to the two reference genes using the qBase software (Biogazelle, Zwijnaarde, BE). The program uses modified software from the classic comparative CT method (ΔΔ CT) that takes into account multiple reference genes and uses inter-run calibration algorithms to correct for run-to-run differences [23]. Coefficient of variation for telomeres was 2.6%, for reference genes 1.6% and for the T/S ratio less than 7%. The corresponding numbers for tissue samples were less than 1% for telomeres and reference genes and 5% for the T/S ratio. Although this assay provides a relative measurement of telomere length, T/S ratios correlate well with absolute telomere lengths determined by Southern blot (r= 0.89, N= 20) [24]. Of the 352 consecutive kidney donors that were eligible for Cohort 1, 217 peripheral blood samples were included; one hundred thirty-five samples were not evaluated, either due to insufficient amount of leucocytic DNA available, or due to insufficient DNA quality for reliable telomere assessment. Accelerated biological aging was evaluated by calculating delta (Δ) biological age - calendar age. Δ (range −3 – 3) was calculated using the difference of quartile telomere length (0 = longer telomere length, 3 = shortest telomere length) - quartile calendar age (0 = youngest age group, 3 = oldest age group).

### Telomere length in kidney biopsies

Since November 2012, next to the wedge biopsy that was used for histological evaluation, a full renal cortical biopsy core was obtained prior to implantation, and immediately stored in Allprotect Tissue Reagent (Qiagen, Venlo, The Netherlands) solution, until extraction. DNA extraction was performed by the Allprep DNA/RNA/miRNA Universal Kit (Qiagen, Venlo, The Netherlands) on a QIAcube instrument (Qiagen, Venlo, The Netherlands). Telomere length in renal tissue samples was measured by a modified version of the protocol for the peripheral blood samples. Telomere length was measured as the telomere repeat copy number relative to a single gene nuclear control genes (*36B4*). The telomere reaction mixture contained 1x Qiagen Quantitect Sybr Green Mastermix 2.5 mM of dithiothreitol, 300 nM of telomere forward primer (5′-ACACTAAGGTTTGGGTTTGGGTTTGGGTTTGGG TTAGTGT-3′), and 900 nM of telomere reverse primer (5′-TGTTAGGTATCCCTATCCCTATCCCTATCCC TATCCCTAACA-3′). The PCR ran for 1 cycle at 95°C for 10 min for activation of the DNA polymerase, followed by 2 cycles of 15 sec at 94°C and 2 min at 49°C, and 30 cycles of 15 sec at 94°C, 20 sec at 62°C and 1 min 40 sec at 74°C. All 40 biopsies included in Cohort 2 passed quality control for assessment of intrarenal telomere length.

### Statistical analysis

Differences between groups were analyzed using parametric or non-parametric one-way ANOVA. Associations between the clinical demographics, telomere length and renal histology were assessed by means of linear or logistic regression analysis, as appropriate, as well as with principal component analysis. From the principal component analysis, a two-dimensional scatter plot was generated to represent the histologic lesions according to their score in the loading matrix. Multiple linear and binomial logistic regressions, with backward parameter selection, were used to model the determinants of telomere length. For backward parameter selection, the following variables were considered for entry into the model for the determinants of telomere length: calendar age, gender, history of hypertension, history of diabetes mellitus and body mass index, history of cardiovascular events, living vs. deceased donor, brain-death vs. cardiac death, ischemic stroke as reason for death, hemorrhagic stroke as reason for death and renal function (eGFR (mL/min/1.73m2). The associations between calendar age, telomere length and the different histological lesions were adjusted for gender and history of cardiovascular events in the multiple logistic regression analyses. All tests were two-sided and *p* values of less than 0.05 were considered to indicate statistical significance. The results are expressed as numerical values and percentages for categorical variables and as mean ± standard deviation for continuous variables, unless otherwise specified. Analyses were done with SAS (version 9.2; SAS institute, Cary, NC), JMP9.0 (SAS institute, Cary, NC) and GraphPad Prism (version 5.00; GraphPad Software, San Diego, CA) software.
